# Supplementary material for: Resilin Distribution and Abundance in Apis mellifera across Biological Age Classes and Castes
Source: Insects. 2023 Sep 13;14(9):764. doi: 10.3390/insects14090764 (PMC10532044; doi:10.3390/insects14090764)
Supplement: Supplementary file 1 [file insects-14-00764-s001.zip › insects-2490132-supplementary.pdf]

Supplementary Material

Table S1. Pro-resilin gene expression pairwise comparisons

Groups	d	Z	P
Pupae:Hatchling	1.579546314	4.116963	0.001
Pupae:Nurse	1.594999201	3.954266	0.001
Pupae:Forager	1.576622853	4.289293	0.001
Pupae:Drone	1.584572668	4.057029	0.001
Hatchling:Nurse	0.015452888	-1.772973	0.957
Hatchling:Forager	0.002923461	-2.183139	0.990
Hatchling:Drone	0.005026354	-2.112737	0.985
Nurse:Forager	0.018376348	-1.658888	0.947
Nurse:Drone	0.010426533	-1.854225	0.964
Forager: Drone	0.007949815	-1.874002	0.966

Table S2. Resilin autofluorescence pairwise comparisons

Joint	Comparison	Difference	UCL (95%)	Z	Pr > d
**1m-cu LFV**	**Hatchling:Nest**	**57263.304**	**25317.4455**	**3.44131179**	**0.001**
**1m-cu LFV**	**Hatchling:Forager**	**69035.7392**	**28250.146**	**3.49431687**	**0.001**
**1m-cu LFV**	**Hatchling:Drone**	**46930.0498**	**29836.1852**	**2.46024538**	**0.004**
1m-cu LFV	Nest:Forager	11772.4352	25275.9272	0.32364323	0.404
1m-cu LFV	Nest:Drone	10333.2542	29037.0123	0.03615677	0.505
1m-cu LFV	Forager:Drone	22105.6894	31876.7721	0.94777065	0.186
**1m-cu RFV**	**Hatchling:Nest**	**70324.6618**	**28806.2814**	**3.60452215**	**0.001**
**1m-cu RFV**	**Hatchling:Forager**	**93316.7512**	**33453.9762**	**4.02039391**	**0.001**
**1m-cu RFV**	**Hatchling:Drone**	**55003.0397**	**37371.7886**	**2.46272979**	**0.003**
1m-cu RFV	Nest:Forager	22992.0894	30558.4961	1.13748583	0.13
1m-cu RFV	Nest:Drone	15321.6221	33072.8736	0.32977315	0.388
**1m-cu RFV**	**Forager:Drone**	**38313.7115**	**35935.828**	**1.63747421**	**0.037**
**Cu-V LFV**	**Hatchling:Nest**	**132753.223**	**38658.5587**	**4.29623782**	**0.001**
**Cu-V LFV**	**Hatchling:Forager**	**114969.295**	**45917.0984**	**3.53513115**	**0.001**

**Cu-V LFV**	**Hatchling:Drone**	**96066.7241**	**48502.4515**	**2.90827601**	**0.001**
Cu-V LFV	Nest:Forager	17783.9283	42986.5627	0.2621798	0.422
Cu-V LFV	Nest:Drone	36686.4993	43121.9875	1.30343222	0.096
Cu-V LFV	Forager:Drone	18902.571	50758.1927	0.1068824	0.476
**Cu-V RFV**	**Hatchling:Nest**	**133513.282**	**36701.8088**	**4.58908456**	**0.001**
**Cu-V RFV**	**Hatchling:Forager**	**101749.066**	**40945.001**	**3.59836823**	**0.001**
**Cu-V RFV**	**Hatchling:Drone**	**116430.4**	**45739.336**	**3.55974306**	**0.001**
Cu-V RFV	Nest:Forager	31764.2157	35935.2966	1.357537	0.091
Cu-V RFV	Nest:Drone	17082.8812	43324.9488	0.21792428	0.436
Cu-V RFV	Forager:Drone	14681.3346	46821.7216	-0.0686192	0.544
**CU-V LHV**	**Hatchling:Nest**	**77861.741**	**31073.9558**	**3.62298199**	**0.001**
**CU-V LHV**	**Hatchling:Forager**	**53534.4025**	**39401.6163**	**2.18067995**	**0.005**
**CU-V LHV**	**Hatchling:Drone**	**44920.1757**	**40569.8623**	**1.72569372**	**0.032**
CU-V LHV	Nest:Forager	24327.3385	34541.9796	0.91268266	0.183
CU-V LHV	Nest:Drone	32941.5653	39123.5341	1.27268412	0.092
CU-V LHV	Forager:Drone	8614.22684	45284.1371	-0.5292954	0.684
**CU-V RHV**	**Hatchling:Nest**	**80965.8069**	**35889.0299**	**3.5164543**	**0.001**
**CU-V RHV**	**Hatchling:Forager**	**60009.609**	**39455.4482**	**2.39721158**	**0.004**
**CU-V RHV**	**Hatchling:Drone**	**49871.8772**	**44595.6124**	**1.80728645**	**0.027**
CU-V RHV	Nest:Forager	20956.1978	39538.5656	0.57686996	0.303
CU-V RHV	Nest:Drone	31093.9296	42354.8413	1.02296662	0.169
CU-V RHV	Forager:Drone	10137.7318	49371.2441	-0.4439794	0.673
